# Incidence, Risk Factors, and Outcomes of Thrombocytopenia in Older Medical Inpatients: A Prospective Cohort Study

**DOI:** 10.3390/hematolrep16040076

**Published:** 2024-12-13

**Authors:** Ioanna Papakitsou, Andria Papazachariou, Theodosios D Filippatos, Petros Ioannou

**Affiliations:** 1 Department of Internal Medicine, University Hospital of Heraklion, 71500 Heraklion, Greece; medp2011875@med.uoc.gr (I.P.); filtheo@uoc.gr (T.D.F.); 2 School of Medicine, University of Crete, 71003 Heraklion, Greece

**Keywords:** thrombocytopenia, older people, medical inpatients, hospitalized, mortality

## Abstract

Background: Thrombocytopenia, defined as a platelet count of less than 150 × 10^9^/L, is a frequent condition among hospitalized patients and presents unique challenges in diagnosis and management. Despite its commonality, data on incidence and related risk factors in medical inpatients remain limited, especially in older people. Methods: A 2-year prospective cohort study with a 3-year follow-up was conducted on inpatients aged ≥65 years admitted to a medical ward. Clinical data were collected, including demographics, comorbidities, laboratory results, and outcomes. Multivariate logistic regression analysis assessed risk factors associated with non-resolution of thrombocytopenia and mortality. Results: The study included 961 older inpatients with a mean age of 82 years. Thrombocytopenia occurred in 22.6% of the study population. The most common causes were infections (57.4%) and drug-induced thrombocytopenia (25.3%). The non-resolution of thrombocytopenia was noted in 59% of patients. In-hospital and 3-year mortality was significantly higher in this subgroup compared to the rest (24.5% vs. 12.7%, *p* = 0.015) and (72.4% vs. 59.8%, *p* = 0.04, respectively). In multivariate analysis, nadir platelet count and hematologic disease were independent factors associated with the non-resolution of thrombocytopenia. Furthermore, in individuals with thrombocytopenia, the administration of norepinephrine (*p* < 0.001) and a higher clinical frailty score (*p* < 0.001) were observed as independent mortality predictors. Conclusions: Thrombocytopenia in older medical inpatients is associated with poor prognosis, particularly in those with non-resolution thrombocytopenia. Early identification and targeted management may improve outcomes.

## 1. Introduction

Thrombocytopenia, defined as a platelet (PLT) count of less than 150 × 10^9^/L, is a common condition affecting up to 50% of hospitalized patients and is characterized by a variable clinical expression [1]. In acute care hospitals, the incidence of thrombocytopenia is about 1% of adult inpatients. Its severity is based on the PLT count: mild (100−150 × 10^9^/L, moderate (50−100 × 10^9^/L and severe <50 × 10^9^/L [2]. It is predominately a secondary condition often identified incidentally during laboratory examination. There can be multiple triggers, including sepsis, severe infection, trauma, toxins, malnutrition, and malignancies; therefore, its diagnosis can be challenging [3].

Although the morbidity and mortality associated with thrombocytopenia are generally low in medical inpatients, its presence frequently indicates a poor prognosis due to its association with underlying disorders, particularly in intensive care unit (ICU) patients [4]. In the ICU, several significant factors were identified that contribute to the onset of thrombocytopenia, including the severity of illness, drugs, organ dysfunction, sepsis, septic shock, and renal failure [5]. These patients face an elevated risk of bleeding and experience extended length of stay and an increased risk of mortality. Yet, there is a gap in the literature concerning the incidence of thrombocytopenia in medical inpatients, especially those of older age, and its related risk factors [6,7].

This cohort study aims to investigate the incidence of thrombocytopenia in a hospitalized population of older age in medical wards and identify factors associated with the non-resolution of thrombocytopenia and in-hospital and long-term mortality.

## 2. Materials and Methods

### 2.1. Study Population

This prospective cohort study evaluated older patients with thrombocytopenia admitted to the Department of Internal Medicine of the University Hospital of Heraklion, Greece. Inclusion criteria were age equal to or greater than 65 years old (based on the World Health Organization (WHO) definition of older age, [8]) and the diagnosis of thrombocytopenia on admission or during hospitalization. Thrombocytopenia was defined as PLTs < 150,000 and classified based on its severity into three groups: mild (PLTs: 100–150,000), moderate (PLTs: 50,000–100,000), and severe (PLTs < 50,000) [1]. Data recorded and evaluated included demographics [age, sex, weight, height, body mass index (BMI)], the cause of admission, past medical history [chronic heart failure (HF), coronary artery disease (CAD), diabetes mellitus (DM), hypertension, chronic kidney disease (CKD), chronic liver disease, chronic lung disease, hematologic diseases], chronic medication use, a medication known to predispose to thrombocytopenia, laboratory exams [(complete blood count (CBC), liver function tests, serum creatinine (Cr), disseminated intravascular coagulation (DIC) screen [fibrinogen, d-dimer, international normalized ratio (INR), partial thromboplastin time, lactate dehydrogenase], vitamin B12 levels (B12) and albumin levels, causes of thrombocytopenia, and data regarding microbiology and serology. Norepinephrine was administered to patients with hemodynamic instability due to either septic shock or hypovolemic shock. Patients were followed for three years post-discharge. Endpoints of interest included in-hospital death, transfer to the ICU, the duration of thrombocytopenia, median length of stay, 3-year post-discharge death, and readmissions during three years of port discharge. The conduct of this study was approved by the Ethics Committee of the University Hospital of Heraklion, Crete (approval code 716/16-01-2019).

### 2.2. Statistical Methods

Unpaired Student’s *t*-test was used to compare continuous variables with normal distribution, and the Mann–Whitney U test was used for skewed distribution. A chi-square test was utilized to compare the categorical variables. Data were summarized as the mean [standard deviation (SD)], median [interquartile range (IQR)], or percentages. The factors associated with the non-resolution of thrombocytopenia, as identified in the literature, including age, comorbidities (CKD, chronic liver disease, malignancy, autoimmune disorders, CAD, hematologic diseases, dementia), chronic medication use, laboratory parameters (platelet count on admission, nadir platelet count, B12 levels, CBC), frailty scores (Fried five phenotype index, clinical frailty score), functional status scores (Katz index), and the identified cause of thrombocytopenia, were selected for univariate analyses to identify factors related to the non-resolution of thrombocytopenia. Similarly, univariate analyses were conducted to determine mortality predictors in hospitalized patients with thrombocytopenia, utilizing well-established factors including age, comorbidities, the severity of thrombocytopenia (resolution or non-resolution of thrombocytopenia, platelet count on admission, nadir platelet count), B12 levels, administration of norepinephrine, frailty scores, functional status assessments, nursing home residency and the duration of hospitalization. Multivariate logistic regression analyses were then performed on statistically significant univariate variables to identify risk factors for both the non-resolution of thrombocytopenia and mortality. All the above statistics were calculated with SPSS version 25.0 (IBM Corp., Armonk, NY, USA). All tests were two-tailed, and *p*-values < 0.05 were considered significant.

## 3. Results

During the study period, 1100 patients were admitted to the Internal Medicine Ward for more than 48 h. Among these, 961 were over 65 years old, and 259 developed thrombocytopenia either at admission or during hospitalization, as determined by laboratory examinations. Ten individuals were excluded from the analysis due to a diagnosis of pseudo-thrombocytopenia (*n* = 7 with giant platelets, *n* = 3 with platelet satellitism). Consequently, the incidence of thrombocytopenia was 22.6%, and the number evaluated was 249 inpatients. All patients in the study were of Greek ethnicity and Caucasian descent.

The mean age of the evaluated cohort was 82.3 (±7.9 years), with 131 (52.6%) being male and 118 (47.4%) females. Regarding their medical history, the most prevalent comorbidities were hypertension (53.4%), followed by diabetes mellitus (43.8%), chronic heart failure (43.8%), and chronic kidney disease (20.1%). The mean age-adjusted Charlson comorbidity index was 5.7. Additionally, 45.8% were bedridden, and 7.6% were residents of nursing homes. Polypharmacy was present in 69.5% of the cohort. A significant portion of the population was classified as frail: 58.2% according to the Fried Five Phenotype index and 61.7% according to the Clinical Frailty Scale. In-hospital mortality was 19.7%.

Of the 249 patients evaluated, 197 (79.1%) presented with thrombocytopenia upon admission, while the remaining 20.9% developed it during hospitalization. The mean duration of thrombocytopenia was 7.7 (±3.7) days. The most common causes of thrombocytopenia were infections (57.4%), followed by possible drug-induced thrombocytopenia (25.3%). Among the infections, 120 cases (84.5%) were attributed to sepsis, 13 cases (9.2%) to influenza, 8 cases (5.6%) to other viral causes, and 1 case (0.7%) to leishmaniasis. An enzyme-linked immunosorbent assay (ELISA) was performed to detect heparin–platelet factor 4 antibodies in seven patients. Three patients tested positive and were diagnosed with immune-mediated heparin-induced thrombocytopenia (HIT). Antibiotics were identified as the cause of thrombocytopenia in 47 patients (74.6%), with piperacillin/tazobactam being the most common (18 patients), followed by trimethoprim/sulfamethoxazole (8 patients), tigecycline (7 patients), linezolid (5 patients), cefepime (5 patients), and ampicillin (4 patients). Although thrombocytopenia related to antibiotic use may coincide with infection development and resolution, the diagnosis of drug-induced thrombocytopenia in this study was confirmed using Naranjo criteria. All patients scored between 1 and 4, indicating a possible association with the implicated antibiotics. A time-dependent decrease in platelet count was observed with drug administration, and recovery upon drug withdrawal was noted in 75% of patients on trimethoprim/sulfamethoxazole, 68% on piperacillin/tazobactam, and 60% on linezolid. Notably, recovery was not observed in 80% of patients receiving tigecycline [9]. Immunosuppressive agents were responsible for thrombocytopenia in seven patients (11.1%). Major bleeding events, including intracerebral hemorrhage, the need for massive blood transfusion, or death due to hemorrhagic shock, were not observed.

The non-resolution of thrombocytopenia was noted in 59% of the cohort. No significant differences were observed between the two groups regarding past medical history, yet the non-resolution group had slightly higher morbidity expressed with a Charlson comorbidity index of 5.9 (±1.2) vs. 5.5 (±1.0) (*p* = 0.088). In addition, the non-resolution group exhibited a significantly lower median Barthel index of 45.0 (0.0, 95.0) compared to 85.0 (10.0, 100.0) in the PLT resolution group (*p* = 0.027). Additionally, the non-resolution group exhibited a higher mean Fried Frailty Phenotype Index (2.9 ± 1.4) and mean Clinical Frailty Scale (5.7 ± 2.2) compared to the PLT resolution group (2.5 ± 1.5, *p* = 0.030 and 4.8 ± 2.3), *p* = 0.006, respectively).

Patients with the non-resolution of thrombocytopenia had a higher incidence of norepinephrine use (20.4%) compared to the rest (9.8%, *p* = 0.018). In-hospital mortality (24.5% versus 12.7%, *p* = 0.015) and 3-year post-discharge mortality (72.4% versus 59.8%, *p* = 0.040) were also significantly higher in the non-resolution group compared to the PLT resolution group. However, no statistically significant differences were found between the two groups regarding length of stay (marginally non-significant), ICU transfer, or readmission rates. Table 1 presents the demographics and comorbidities, laboratory values, and outcomes of the sample with thrombocytopenia.

No statistically significant difference was observed regarding mean platelet count on admission to the group without the resolution of thrombocytopenia compared to the group with the resolution of thrombocytopenia, 124.0 (±57.0) and 139.0 (±103.0), respectively (*p* = 0.161). However, the mean nadir platelet count in patients with non-resolution of thrombocytopenia was 87.0 (±33.0) compared to 101.0 (±29.0) in those whose thrombocytopenia resolved (*p* < 0.001).

Hospital-acquired thrombocytopenia was present in 18.6% of the group with resolution of thrombocytopenia patients compared to 22.4% in those without the resolution of thrombocytopenia (*p* = 0.285). The group without the resolution of thrombocytopenia also had a higher incidence of severe thrombocytopenia (15.6% vs. 4.9%, *p* = 0.008), while mild thrombocytopenia was more common in the group with the resolution of thrombocytopenia (56.9% vs. 42.4%, *p* = 0.016). Twelve out of twenty-three patients with severe non-resolution of thrombocytopenia were diagnosed with infection, nine with possible drug-induced thrombocytopenia, and two with hematologic malignancy. Additionally, septic shock was more frequent in the group without the resolution of thrombocytopenia (20.4% vs. 9.8%, *p* = 0.018), and that group had a higher prevalence of hematologic diseases (21.1% vs. 6.9%, *p* = 0.001). In this cohort, patients with hematologic diseases were diagnosed with either myelodysplastic syndromes (*n* = 25) or lymphoid malignancies (*n* = 13), including Hodgkin’s lymphoma and mature B-cell neoplasms. Lastly, drug-induced thrombocytopenia was more prevalent in the group with the resolution of thrombocytopenia (31.4% vs. 21.1%, *p* = 0.046). Table 2 presents the PLT count, severity, and causes of thrombocytopenia.

The risk factors associated with the non-resolution of thrombocytopenia in older medical inpatients during hospitalization were identified using multivariate logistic regression analysis. Candidate variables with significant *p*-values in univariate analysis, such as nadir platelet count (*p* < 0.001), hematologic diseases (*p* = 0.003), Katz index (*p* = 0.046), the administration of norepinephrine (*p* = 0.028), dementia (*p* = 0.029), the clinical frailty score (*p* = 0.006), and the Fried five phenotype index (*p* = 0.031) were entered into the model. In multivariate analysis, only nadir platelet count (*p* = 0.004, EXP[B] 1.100, 95% CI: 1.000–1.500) and hematologic disease (*p* = 0.005, EXP[B] 3.561, 95% CI: 1.453–9.729) were independent factors associated with the non-resolution of thrombocytopenia.

In the second analysis in our cohort study, 49 individuals were classified as non-survivors, while the remaining 200 were classified as survivors. Non-survivors had a significantly lower mean platelet count on admission (127.0 ± 78.0 × 10^3^) compared to survivors (144.0 ± 82.0 × 10^3^) (*p* = 0.033). Additionally, the nadir platelet count was notably lower in non-survivors (84.0 ± 38.0 × 10^3^) than in survivors (95.0 ± 31.0 × 10^3^) (*p* = 0.010). Non-survivors were more likely to be nursing home residents, with 16.3% of non-survivors versus 5.5% of survivors (*p* = 0.017). Furthermore, non-survivors had a significantly lower median Barthel index (80.0, IQR 30.0–100.0) compared to survivors (100.0, IQR 75.0–100.0) (*p* < 0.001). The Fried Frailty Index was also higher in non-survivors, with a mean score of 3.7 (±1.0) compared to 2.5 (±1.5) in survivors (*p* < 0.001), as was the Clinical Frailty Index, which averaged 7.0 (±1.5) in non-survivors versus 3.7 (±1.0) in survivors (*p* < 0.001).

A logistic regression analysis was conducted to identify factors associated with in-hospital mortality in individuals who developed thrombocytopenia, as detailed in Table 3. The univariate logistic regression analysis identified nadir platelet count (*p* = 0.0041), sepsis (*p* = 0.003), a higher Katz index (*p* < 0.001), dementia (*p* = 0.014), nursing home residency (*p* = 0.015), bed-ridden status (*p* = 0.003), a higher Fried Five phenotype index (*p* < 0.001) and higher clinical frailty score, which were positively associated with in-hospital mortality. Conversely, the resolution of thrombocytopenia (*p* = 0.024) and the higher Barthel index (*p* < 0.001) were negatively associated with mortality. The multivariate logistic regression analysis revealed that only the administration of norepinephrine (*p* < 0.001) and a higher clinical frailty score (*p* < 0.001) were independent factors associated with mortality.

## 4. Discussion

To our knowledge, this prospective cohort study is the first to document the incidence and etiologies of thrombocytopenia and the risk factors associated with the persistence of thrombocytopenia and mortality in an older medical inpatient population. Most of the patients were very old with a high Charlson comorbidity index, indicating a >85% 1-year mortality rate [10]. Approximately one out of every five patients died during hospitalization, and two out of three died during the 3-year follow-up. Over half of the patients were readmitted to the hospital for any cause during the follow-up period. The elevated mortality and readmission rates observed in this cohort of inpatients with thrombocytopenia could be attributed to the inclusion of a very older population characterized by significant morbidity and frailty.

The incidence of thrombocytopenia in our medical ward was 22.6%. Hospital-acquired thrombocytopenia was presented in 1 to 5 patients. The literature has conflicting data regarding the incidence of thrombocytopenia; according to previous studies evaluating thrombocytopenia in medical wards, the reported incidence varies from 6.3% to 18.7% [11,12,13]. The substantial variation in thrombocytopenia incidence is likely attributable to the diverse characteristics of the study populations, coupled with differing criteria for their definition and underlying etiologies. For instance, in a study on medical inpatients, although definition levels were the same as ours, patients with malignancy or ICU transfer were excluded [11]. In another study in 2076 patients, the prevalence was 14.9%, yet the thrombocytopenia definition was a platelet count of less than 100 × 10^9^/L [13]. A higher incidence ranging from 15 to 53% is reported in studies including critically ill patients from ICU [7,14,15,16,17].

Non-resolution thrombocytopenia was noted in 59% of the cohort. Compared to the rest, these individuals experienced higher rates of administration of norepinephrine, in-hospital mortality, and 3-year post-discharge mortality. The results corroborate with the conclusions drawn in other research, where the non-resolution of thrombocytopenia associated with mortality was similarly identified. In a retrospective analysis of patients admitted with sepsis/septic shock in the ICU, those with the non-resolution of thrombocytopenia had higher rates of mortality. In a multivariate analysis adjusted for age, APACHE III score, and compliance with a sepsis resuscitation bundle, the non-resolution of thrombocytopenia was significantly associated with 28-day mortality [18]. Another retrospective worldwide study from Europe, America, and Australia, examining the progression of platelet counts in critically ill patients, found that mortality rates can reach 66% when thrombocytopenia persists for 14 days after initial ICU admissions. In contrast, mortality drops to 16% if platelet levels are successfully restored [16]. Drug-induced thrombocytopenia presented higher rates in those with a resolution of thrombocytopenia compared to the rest. Similar patterns have been documented in other studies [19,20] since the discontinuation of the causative medication typically reverses thrombocytopenia. Unlike thrombocytopenia caused by the more severe or non-resolution of underlying conditions such as sepsis or hematologic malignancies, drug-induced cases generally improve relatively quickly with appropriate intervention, resulting in a shorter duration of thrombocytopenia and a higher likelihood of resolution. According to a review, typically, recovery from drug-induced thrombocytopenia begins within 1 to 2 days after discontinuing the offending drug, with complete recovery usually occurring within a week [21].

Νon-survivors had a longer duration of thrombocytopenia, lower mean platelet counts on admission, and lower mean nadir platelet count. Similar results were observed in a study in the ICU population, where mortality was higher in patients with a nadir platelet count of <100.000/mL and death rates were increasing from mild to very severe thrombocytopenia compared to those without thrombocytopenia [22]. Furthermore, in a retrospective cohort study evaluating patients with thrombocytopenia and infectious diseases, after defining three subgroups according to the nadir platelet count within seven days, the group with the lower platelet count was associated with higher ICU mortality [23]. The association between the duration of thrombocytopenia and increased mortality is well-prescribed in the literature only in ICU patients [22,24,25,26,27]. A retrospective study in surgically critically ill patients observed that among patients with non-resolution of thrombocytopenia, mortality was significantly higher [26]. The prolonged period of thrombocytopenia can be associated with mortality since it often indicates a more severe or non-resolution of underlying pathology. For instance, a study of 3291 patients in the ICU revealed that as the duration of thrombocytopenia increased, disease severity was higher, and higher rates of vasoactive agents were received, as well as undergoing renal replacement therapy [27]. Non-survivors are generally more critically ill, which can lead to more profound and sustained platelet consumption or even bone marrow suppression, such as sepsis, certain medications, or underlying conditions (e.g., malignancies) [28,29]. Furthermore, in this study, non-survivors with thrombocytopenia had a lower Barthel index and higher Fried and clinical frailty scale. Frailty is associated with multiple adverse health outcomes [30], and it is reasonable that they have higher mortality rates; however, frail individuals may have diminished physiological reserve or compromised health status, making the recovery of platelet counts challenging [31].

In our cohort, infection emerged as a leading cause of thrombocytopenia, accounting for over half of the cases. Among these, sepsis was identified in approximately 85% of the affected patients. Our findings are consistent with those reported in previous studies observing the causes of thrombocytopenia in hospitalized patients. According to a single-institutional retrospective study in a total of 711 patients, infection was documented in 56% of all patients with thrombocytopenia [11]. In another retrospective study involving ICU patients with thrombocytopenia, infection was reported at a lower rate of 32.7%, while sepsis was observed in 3.93% of cases [32]. Additionally, an observational cohort study of 105 consecutive patients admitted to the ICU with sepsis/septic shock in Greece indicated an incidence of thrombocytopenia in 53% of patients at the time of admission [17]. Sepsis-associated thrombocytopenia has several possible mechanisms, such as the decreased production of platelets, interactions between platelet receptors, immune-associated thrombocytopenia, platelet sequestration, and consumptive coagulopathy [29]. Given the high incidence of thrombocytopenia associated with sepsis and its recognition in the literature as both a prognostic marker and an independent predictor of poor outcomes [18,33,34], careful monitoring is essential, with close attention to platelet counts and the timing of thrombocytopenia onset [5].

Drug-induced thrombocytopenia was observed in one out of four patients, with antibiotics accounting for nearly three-quarters of the cases. The most common antibiotics in this cohort were piperacillin/tazobactam and trimethoprim/sulfamethoxazole. Among the causes of thrombocytopenia, drugs are often suspected when more apparent causes have been excluded [35]. To our knowledge, the incidence of drug-induced thrombocytopenia in medical inpatients is not well-documented in the literature. However, it is estimated to affect between 10 and 25% of critically ill patients [36,37]. This variation may be attributed to the fact that, in most cases, drug etiology is determined based on clinical criteria rather than through the demonstration of drug-dependent antiplatelet antibodies [21]. Commonly used antibiotics, such as extended-spectrum b-lactams, cephalosporins, quinolones, carbapenems, and linezolid, have been associated with drug-induced thrombocytopenia [38,39,40,41,42]. The associated mechanisms are either an increased destruction due to immune-mediated thrombocytopenia or a decrease in platelet production due to bone marrow toxicity [38]. In this cohort, the duration of thrombocytopenia associated with piperacillin/tazobactam had a mean of four days, while for trimethoprim/sulfamethoxazole, the mean duration was six days. Regarding piperacillin/tazobactam, almost all case reports describe rapid-drug-induced thrombocytopenia [41,43,44,45], with immediate improvement following the withdrawal of the antibiotic and, in certain cases, with the initiation of steroids and intravenous immunoglobulins.

Finally, our findings provide valuable insights into the factors associated with the non-resolution of thrombocytopenia and in-hospital mortality among older medical inpatients. Lower nadir platelet counts and the presence of hematologic malignancies were identified as independent predictors of the non-resolution of thrombocytopenia, highlighting the need for the close monitoring of patients with these risk factors to potentially reduce the duration and impact of thrombocytopenia. Monitoring platelet trends during hospitalization is crucial, particularly for older patients with infection, sepsis, septic shock, polypharmacy, or suspected drug-induced thrombocytopenia. A drop in the platelet count within 24 h is usually dilutional. The use of peripheral smear is crucial for excluding pseudo thrombocytopenia and accurately determining the true platelet count. Ruling out life-threatening causes such as sepsis-, heparin- or drug-induced thrombocytopenia, and hematologic diseases like acute leukemia is the most important step. The early identification of infections and sepsis with appropriate antimicrobial therapy based on local antibiograms is critical to mitigating infectious causes of thrombocytopenia. Prompt hematology consultation for advanced diagnostics in suspected hematological malignancy or for managing established disease under targeted treatment is highly beneficial.

Furthermore, norepinephrine and a higher Clinical Frailty Score were identified as independent predictors of mortality, underscoring the critical influence of frailty and illness severity on patient outcomes. Our results are in line with the existing literature, further confirming that frailty is a powerful predictor of mortality in older adults regardless of the specific frailty scale [30,46,47]. Incorporating tools like the Clinical Frailty Score and Fried Frailty Phenotype Index into the routine assessment of older medical inpatients can help stratify those at higher risk of complications, including the non-resolution of thrombocytopenia.

This study has certain limitations. This study was conducted at a single tertiary care hospital, which may limit the generalizability of the findings. Although patients were followed for three years post-discharge, other relevant long-term complications associated with thrombocytopenia, or its causes may have been missed due to the natural aging process and high comorbidity burden in this population. Furthermore, while minor bleeding events such as petechiae and ecchymoses are common in older adults, they were not included in this study due to the potential for confounding factors. In this population, these minor bleeds can occur even in the absence of thrombocytopenia. Including them could lead to unreliable data, making it challenging to differentiate between cases directly caused by thrombocytopenia and those induced by other factors. Therefore, the focus of this study remained on major bleeding events. Moreover, a limitation of this study is the potential confounding effect of sepsis in the association between thrombocytopenia and in-hospital mortality. Although we found that infection was the most common cause of thrombocytopenia, it is important to clarify that a large proportion of these cases are associated with sepsis, which is the main risk factor of in-hospital mortality. Thus, the precise role of thrombocytopenia as a prognostic marker in the context of sepsis warrants further investigation in future studies. Long-term platelet recovery data would be useful to better assess outcomes. In addition, including only older patients with thrombocytopenia in the study may have introduced selection bias, as this population may have underlying conditions that could influence both the development and outcomes of thrombocytopenia. Although our study provides valuable insights into thrombocytopenia in older medical inpatients, the findings are limited to a single-center hospital in Greece, which may affect their generalizability. However, the University General Hospital of Heraklion is the largest of five hospitals in Crete, the fifth largest island in the Mediterranean Sea, and serves a substantial patient population, which adds some breadth to the findings.

Future studies should focus on further elucidating the role of thrombocytopenia as a prognostic marker in older medical patients, especially in the context of infections and sepsis. In addition, investigating the impact of targeted therapeutic strategies aimed at improving platelet count recovery in older patients would be valuable. Moreover, exploring the potential for personalized medicine, where frailty and comorbidities influence treatment decisions, could improve patient outcomes. Longitudinal, multicenter studies examining the long-term effects of thrombocytopenia, such as functional impairment and quality of life, would provide deeper insights into the broader consequences of this condition in the aging population.

## 5. Conclusions

This study provides several important observations: One out of five older medical inpatients experienced thrombocytopenia, and non-resolution was observed in 59%. No resolution of thrombocytopenia was significantly associated with higher in-hospital and long-term mortality, emphasizing the clinical importance of monitoring and managing thrombocytopenia, particularly in older patients. Lower nadir platelet counts and those with underlying hematologic malignancy are likelier to experience the non-resolution of thrombocytopenia. Further research is necessary to explore targeted therapies and interventions that could improve the resolution rates of thrombocytopenia and enhance survival.

## Figures and Tables

**Table 1:** Demographics, clinical characteristics, and laboratory values of 249 medical inpatients with thrombocytopenia.

	All Patients (*n* = 249)	Non-Resolution of Thrombocytopenia (*n* = 147)	Resolution of Thrombocytopenia (*n* = 102)	*p*-Value
Demographics and comorbidities				
Age (years), mean (SD)	82.3 (±7.9)	83.2 (±7.8)	81.2 (±8.0)	0.059
Male, *n* (%)	131 (52.6)	79 (53.7)	52 (51.0)	0.700
Female, *n* (%)	118 (47.7)	68 (46.2)	51 (50.0)	0.675
Coronary artery disease, *n* (%)	46 (18.5)	28 (19.0)	18 (17.6)	0.869
Diabetes mellitus, *n* (%)	109 (43.8)	67 (45.6)	42 (41.2)	0.518
Chronic heart failure, *n* (%)	109 (43.8)	66 (44.9)	43 (42.2)	0.698
Hypertension, *n* (%)	133 (53.4)	85 (57.8)	48 (47.1)	0.121
Chronic kidney disease, *n* (%)	50 (20.1)	28 (19.0)	22 (21.6)	0.633
Chronic liver disease, *n* (%)	5 (2.0	2 (1.4)	3 (2.9)	0.403
Chronic lung disease, *n* (%)	30 (12.0)	21 (14.3)	9 (8.8)	0.237
Age adjusted CCI, mean (SD)	5.7 (±1.9)	5.9 (±1.9)	5.5 (±2.0)	0.088
Nursing home inmate, *n* (%)	19 (7.6)	13 (8.8)	6 (5.9)	0.471
Bedridden status, *n* (%)	114 (45.8)	74 (50.3)	40 (39.2)	0.093
Polypharmacy, *n* (%)	173 (69.5)	101 (68.7)	72 (70.6)	0.781
Barthel index, median (IQR)	75.0 (10.0, 100.0)	45.0 (0.0, 95.0)	85.0 (10.0, 100.0)	0.027
Fried frailty phenotype index, mean (SD)	2.7 (±1.5)	2.9 (±1.4)	2.5 (±1.5)	0.030
Clinical Frailty Scale, mean (SD)	5.3 (±2.3)	5.7 (±2.2)	4.8 (±2.3)	0.006
Laboratory values				
Albumin levels g/dL, mean (SD)	3.3 (±0.6)	3.2 (±0.6)	3.3 (±0.6)	0.452
Hemoglobin g/dL, mean (SD)	11.8 (±6.7)	12.2 (±8.6)	11.5 (±2.5)	0.459
Vitamin B12 levels pg/mL, mean (SD)	714.7 (±583.0)	688.6 (±571.2)	716 (±584.9)	0.721
Serum creatinine levels mg/dL, mean (SD)	1.4 (±0.8)	1.4 (±0.7)	1.4 (±1.0)	0.530
Outcomes				
Median length of stay, median (IQR)	8.0 (5.0, 13.0)	7.0 (4.0, 12.0)	9.0 (6.0, 13.0)	0.059
Administration of norepinephrine, *n* (%)	40 (16.1)	30 (20.4)	10 (9.8)	0.018
ICU admission, *n* (%)	5 (2.0)	4 (2.7)	1 (1.0)	0.318
In-hospital mortality, *n* (%)	49 (19.7)	36 (24.5)	13 (12.7)	0.015
3-year post-discharge readmission rates, *n* (%)	124 (50.2)	67 (45.9)	57 (56.4)	0.121
3-year post-discharge mortality, *n* (%)	166 (67.2)	105 (72.4)	61 (59.8)	0.040

CCI: Charlson comorbidity index; ICU: intensive care unit; IQR: interquartile range; SD: standard deviation.

**Table 2:** Platelet (PLT) count, severity, and causes of thrombocytopenia.

Variable	All Patients (*n* = 249)	Non-Resolution of Thrombocytopenia (*n* = 147)	Resolution of Thrombocytopenia (*n* = 102)	*p*-Value
PLT × 10^3^, mean (SD)	130. 0 (±71.0)	124 (±57.0)	139 (±103.0)	0.161
Nadir PLT × 10^3^, mean (SD)	93.0 (±33.0)	87.0(±33.0)	101.0 (±29.0)	<0.001
MPV, mean (SD)	9.7 (±1.3)	9.6 (±1.4)	9.8 (±1.2)	0.273
Severe thrombocytopenia, *n* (%)	28 (11.2)	23 (15.6)	5 (4.9)	0.008
Moderate thrombocytopenia, *n* (%)	101 (40.6)	62 (42.2)	39 (38.2)	0.312
Mild thrombocytopenia, *n* (%)	120 (48.2)	62 (42.4)	58 (56.9)	0.016
Hospital acquired thrombocytopenia, *n* (%)	52 (20.9)	33 (22.4)	19 (18.6)	0.285
Infection - Sepsis, *n* (%) - Septic shock, *n* (%)	194 (77.9) 120 (48.2) 40 (16.1)	114 (77.6) 70 (47.6) 30 (20.4)	80 (78.4) 50 (49.0) 10 (9.8)	1.000 0.465 0.018
Drug-induced thrombocytopenia, *n* (%)	63 (25.3)	31 (21.1)	32 (31.4)	0.046
HIT, *n* (%)	3 (1.2)	2 (1.4)	1 (1.0)	1.000
Liver disease, *n* (%)	13 (5.2)	9 (6.1)	4 (3.9)	0.322
Nutrient deficiencies, *n* (%)	22 (8.8)	14 (9.5)	8 (7.8)	0.413
Hematologic diseases, *n* (%)	38 (15.3)	31 (21.1)	7 (6.9)	0.001
Malignancy-Bone marrow infiltration, *n* (%)	15 (6.0)	10 (6.8)	5 (4.9)	0.369
No diagnosis, *n* (%)	16 (6.4)	10 (6.8)	6 (5.9)	1.000

HIT: heparin-induced thrombocytopenia; MPV: mean platelet volume; PLT: platelets; SD: standard deviation.

**Table 3:** Multivariate logistic regression model analyzing various risk factors for in-hospital mortality in hospitalized patients who developed thrombocytopenia.

Univariate Analyses	OR	CI 95%	*p*-Value
Nadir PLT count	1.020	1.000, 1.020	0.041
Resolution of thrombocytopenia	0.450	0.225, 0.900	0.024
Sepsis	2.679	1.386, 5.179	0.003
Katz index	0.758	0.661, 0.869	<0.001
Dementia	2.312	1.188, 4.503	0.014
Nursing home inmate	3.353	1.269, 8.855	0.015
Bed-ridden status	2.709	1.411, 5.200	0.003
Barthel index	0.983	0.975, 0.991	<0.001
Fried Five Phenotype index	1.901	1.438, 2.513	<0.001
Clinical frailty score	1.696	1.387, 2.073	<0.001
Administration of norepinephrine	15.019	6.878, 32.792	<0.001
Multivariate analysis	OR	CI 95%	*p*-value
Clinical frailty	3.375	1.878, 7.429	<0.001
Administration of norepinephrine	11.625	4.537, 29.786	<0.001

CI: confidence interval; OR: odds ratio; PLT: platelets.

## Data Availability

The data presented in this study are available on request from the corresponding authors.
